# Introducing of Online Channel and Management Strategy for Green Agri-food Supply Chain based on Pick-Your-Own Operations

**DOI:** 10.3390/ijerph16111990

**Published:** 2019-06-04

**Authors:** Qifan Hu, Qianyun Xu, Bing Xu

**Affiliations:** 1 School of Management, Nanchang University, Nanchang 330031, China; setsail@nit.edu.cn; 2 School of Business Administration, Nanchang Institute of Technology, Nanchang 330099, China; 3 School of Sciences, Nanchang University, Nanchang 330031, China; 18166033028@163.com

**Keywords:** green agri-food, pick-your-own operations, introducing online channels, freshness, cooperation contract

## Abstract

The popularity of e-commerce has impacted traditional retail business. Farmer cooperatives running green agri-food pick-your-own (PYO) farms are facing the choice of whether or not to adopt online channels. PYO operation refers to consumers picking and purchasing the agri-food growing on a farm, and due to it being environmentally-friendly, healthy, and popular, it has been widely adopted by many farm cooperatives. This paper aims to discuss the practicality of introducing online channels to already established PYO farms in the green agri-food supply chain (GASC), who can personally take charge of the online channel or transfer it to one online retailer. Firstly, we constructed the demand functions of green agri-food by putting consumer utility, the freshness of agri-food, and transportation cost into consideration. Secondly, five decision models are built to characterize five operation modes, namely pure PYO mode, self-operated dual-channel mode, decentralized dual-channel mode, centralized dual-channel mode, and contractual cooperation mode. Furthermore, by taking price, demand, and profit with different modes into consideration, we are able to explore the introduction of online channels and green brand construction. Finally, numerical analysis is performed. We found that: (1) introducing an online channel is preferable strategy since the profit of the farmer cooperative in pure PYO mode is always less than the profit of a farmer cooperative in non-self-operated dual-channel modes; (2) the decision of self-operating an online channel is related to the fixed cost of creating a new online channel and the green food brand effect of online channel, and it is the optimal mode in some cases, while the contractual cooperation mode is the optimal mode in the remaining cases; and (3) the green food brand effect of online channels is does not necessarily improve with scale, and the initial freshness has a positive relationship to the profit, demand, and price of farmer cooperatives and online retailers.

## 1. Introduction

Online shopping has brought opportunities and threats to traditional retailing, and PYO farms are no exception. With the development of rural tourism, farmer cooperatives have developed various kinds of farms or orchards for picking, recreation, and leisure [1,2,3], where consumers can purchase and pay for green agri-foods picked by themselves. Compared with the traditional retail model, this pick-your-own (PYO) operation offers lots of advantages. First of all, PYO offers direct transport from farm to table, which effectively reduces the “food miles” in transportation that are used to measure environmental impact in terms of contribution to greenhouse gas emissions [4]. This results in a reduced transportation cost and lower greenhouse gas emissions. Moreover, transport trucks are the main transport mode of agri-foods purchased in traditional ways, which emits more greenhouse gases than other transport modes. Consequently, greenhouse gas emissions from fruits and vegetables purchased in traditional ways are relatively high, accounting for 23% of total CO2e of a household [5]. Therefore, the reduction of “food miles” of agri-foods offered by the PYO model is important. Secondly, agri-foods are perishable, and the freshness degree will decrease with time after harvesting [6,7,8,9] with the loss of water and nutrients [9,10]. Unlike agri-foods which decay on shelves and lose their nutrition while waiting to be purchased, PYO outlets have the fruits or vegetables growing while waiting for harvest. This grants customers access to fresher and heathier agri-foods [11,12]. In addition, consumers can enjoy the PYO experience [2,3,11,12,13], and the picking experience is also a selling point. Finally, PYO operations are also economically profitable for farmers. Farmers no longer need to spend manpower and cost to pick the agri-foods [14]. Furthermore, PYO operations’ on-site sales can reduce transport costs [1,14]. Therefore, PYO operations are an environmentally-friendly, sustainable, and healthily consumption model with many benefits. Nevertheless, PYO has some shortcomings. Consumers have to bear certain transportation costs when they go to PYO farms. Moreover, if consumers fail to find green agri-foods that meet their needs at one PYO farm, they may drive to other PYO farms, which may reduce some of the environmental benefits of PYO. Also, PYO farms are mainly aimed at urban residents or incoming tourists. As for some regional green agri-foods, non-local consumers cannot purchase through PYO farm channels without traveling.

Along with the rapid development of information technology and logistics management, e-commerce has become an important part of people’s lives [15]. Online shopping is favored for its convenience and the provision of dynamic information [16,17,18]. However, is it necessary for PYO farms to introduce online channels? Many kinds of agri-foods can be purchased online in different sale modes. For example, some farmer cooperatives have signed to become contractors with large supermarkets while selling the remaining agri-foods through their own self-operated e-commerce channels [19]. Other retailers sell agri-foods in both self-operated online and offline channels [20]. But are PYO farms characterized by experience suitable for opening online channels? In real life, there exist repeat purchases for many consumers who have purchased green agri-foods on PYO farms. So, the online channel will meet these needs and save consumers transportation costs. Moreover, PYO operations also promote online channels since consumers can get the experience of picking green agri-foods and know about the growing environment, and the size, color, taste of green agri-foods. Experience is the key to the success of online brand [21,22]. Consumers can produce satisfaction and behavioral intentions through experience, so that an online brand relationship can be formed [23]. Moreover, the online channel is conducive to the formation of brand relationship by offline operation, which would not be sufficient by simply establishing web pages and generating traffic [24]. Certainly, both offline and online channels have their advantages and disadvantages. For example, online shopping is convenient, but the waiting time for transportation and distribution is long, and the freshness of green agri-foods will decrease; PYO can ensure freshness, but consumers have to drive to the farm. Hence, PYO operations and online channels complement each other.

This paper will answer the following questions. Should online channels be introduced in the PYO operation supply chain? If so, who should operate and manage the online channels? How to construct a green food brand of the online channel and which mode is the optimal for the farmer cooperative or the green agri-food supply chain (GASC)? This paper will first construct the utility function including the price and freshness of green agri-food, and the transportation cost, then obtain the demand function based on utility function. Secondly, five models will be built to characterize five operation modes of GASC, and study the necessity of introducing online channels for PYO farms. Finally, we discuss green brand construction strategy for online channels, the impact of freshness on GASC, and optimal modes for the farmer cooperative and the GASC.

The rest of this paper is organized as follows. In Section 2, we present the literature review. In Section 3, we describe the problem, associated assumptions, and a benchmark model. In Section 4, we develop dual-channel models. In Section 5, we compare the models and analyze. In Section 6, numerical analysis is presented. In Section 7, we discuss the result. In Section 8, we summarize the research and propose future research directions. All proofs of propositions and corollaries are found in Appendix A.

## 2. Literature Review

There are four streams of literature related to this paper. The first stream focuses on the supply chain of agricultural products. Most studies describe the characteristics of the supply chain of agricultural products on the basis of the freshness [25,26,27], perishability [28], and physical loss [29]. Furthermore, many of these studies investigated the optimal decision making of an agricultural product supply chain either on the pricing [27,28], ordering [27,30,31,32], coordinating [25,26,27,30,31,33], or the effects of effort [25,28,31]. Moreover, given the special structure of the agricultural product supply chain, some studies have examined the optimal decisions of various combinations of stakeholders, such as “company + farmer” [34], “Farmer-supermarket Direct Purchases” [35], and “Firm-cooperative-farmer” [36]. Conversely, few have taken PYO into consideration. 

Kadet et al. concluded that the cost would be relatively high for a farm to carry out PYO operations based on the prevalence of it [2]. Rouquet et al. compared PYO farms and large retail stores from the perspective of experiential logistics [12]. Carpio et al.’s research on North Carolina fruit farms further supported the statement arguing that price is an important factor affecting consumers’ choice between PYO or pre-picking [11]. In addition, PYO farms excel in saving labor costs according to Burnap’s research on the Butternut Farm and ideas and signage strategies for operating and developing PYO farms were proposed [14]. However, Frith raised the concern that due to the lack of skills for PYO customers, agri-products with certain characteristics are more suitable for PYO, such as “long bush life of fruit”, “fruit presentation”, “easily harvested clusters”, “sweetness & flavour”, “freezing quality”, and so on [3]. 

These studies mainly used the perspective of a qualitative point of view or an empirical method to analyze the operating points of PYO operations and the purchasing characteristics of PYO customers. In contrast, this paper will study whether or not to open the online channel when one farmer cooperative performs PYO operation. Similarly, this paper would also take the freshness of agricultural products, as well as the problems of pricing and cooperation, into consideration. Therefore, there is a bright spot in this paper.

The second stream focuses on green supply chain management (GSCM). Since 2010, scholars have drawn more attention to green supply chain management [37]. Laari et al. confirmed the correlation between competitive strategies and green supply chain management strategies by empirical method [38]. Zaid et al. investigated the relationship between green human resource management, green supply chain management practices, and sustainable performance in Palestine based on empirical methods [39]. They all used empirical methods. While Tseng et al. reviewed the literature and found that the trend of establishing mathematical optimization model to improve decision-making ability and pursue environmental performance is becoming more and more obvious [37]. In many applied mathematical optimization models, greenness or greening levels are considered as decision variables or research objects [40,41,42,43,44]. Different from them, this paper studies the introduction and green brand management of online channel in GASC.

The third stream is about online brand experience and relationship. Many studies emphasize the importance of consumer experience for online brands [16,22,45]. Morgan-Thomas found that consumer experience is beneficial to the formation of online brand relationship [23]. These studies are all aimed at the single object of online brand or online brand relationship. However, with the development of O2O, online channels do not exist independently in many business models. The self-image congruity and online–offline brand image congruity have an impact on customers’ online brand experiences and trust of the hotel’s brand, and the online channel is conducive to the formation of brand relationship offline operation [24]. Unlike their research, this paper focuses on whether or not to introduce online channels, and discuss if so, how to manage the green agri-food brand of online channel.

The last stream is about channel introduction and management of the supply chain. Some papers discuss such issues in dual-channel and multi-channel supply chains regarding whether an online channel should be introduced, whether online and offline prices should be unified, how to address channel conflicts, and which contracts are effective [46,47], based on the optimal pricing value, optimal order value, or other optimal requirements. Hernant et al. used empirical research to find that when offline customers become cross-channel customers, their offline frequency and regularity will change. This change will affect offline sales and operations. Therefore, they suggest that offline retailers should be careful when opening online channels [48]. Whereas others discussed this issue based on the modeling approach. Shi et al. used the physical option approach to study the correct time for the introduction of the online channel while operating costs remain stochastic [49]. Zhang et al. studied the channel structure selection and pricing strategies in a supply chain consisting of a retailer and a manufacturer, and concluded that the channel structure selection of the retailer is affected by consumer acceptance rate of online channel [50]. Chen et al. studied the decision-making of online channel introduction under the environment of high return rate of online channel, and they found that the choice of an online channel, offline channel, or dual channel model depends on the efficiency ratio of the online channel and offline channel [51]. Nie et al. studied the decision-making of whether to open an online channel when two offline retailers compete, and found that the significant and positive and negative of cross-channel effects have an impact on the decision-making [52]. The above-mentioned study discusses whether traditional retailers should open online channels, while this paper discusses whether online channels should be introduced by farmer cooperative. There is research closely related to this paper. Li et al. investigated a manufacturer with traditional retail channel about the introduction of the online channel and the brand, and found that brand introduction is appropriate, while the introduction of an online channel depends on the situation [53]. In contrast, their research subject is a manufacturer who sell products through an offline retailer, while ours is the farmer cooperative that provides consumers with PYO operations similar to direct sale. Moreover, what they want to know is whether to introduce a new brand. We study the management strategy of online green agri-food branding. 

## 3. Model Description and Benchmark Model

### 3.1. Problem Description

**Figure 1:**
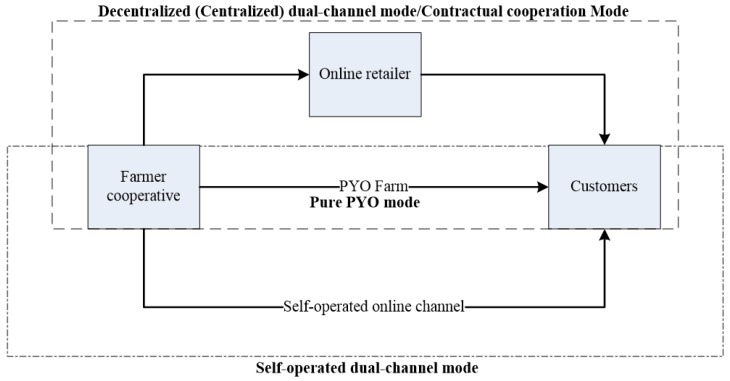
Problem structure.

A farmer cooperative who operates a PYO farm for green agri-food, tries to introduce an online sale channel. There are two ways to operate the online channel—a self-operated dual-channel run by the farmer cooperative or authorizing an online retailer to sell green agri-food. In first way, the farmer cooperative needs to build a new online shopping platform and operate the online channel by itself. In second approach, the farmer cooperative authorizes an online retailer to sell green agri-food. Based on the principle of maximizing profits, the farmer cooperative decides whether to introduce an online channel or not, and which way to operate the online channel when introducing an online channel. The structure of the problem is shown in Figure 1. When online channel is introduced, consumers can obtain green agri-food by two channels of shopping: PYO and direct purchase (denoted by f), online purchase and offline distribution (denoted by o). Consumers have different value assessments for green agri-food in channel *i*, which is denoted as $\nu_{i}(i=f,o)$. On the PYO farm, consumers can touch and taste green agri-food, and then choose their desired green agri-food from size, shape and other aspects. However, in the online channel, consumers can not see and touch the green agri-food they want to purchase in advance, nor can they try them in advance. They mainly rely on the online channel of pictures, text descriptions, and other brand effects to understand the products. As a result, consumers’ expectations of the utility of online green agri-food have been reduced. Since consumers have more expectations of the utility in a PYO channel than in an online channel. Assume $\nu_{f}=\nu$, and $\nu_{o}=\alpha \nu$ (0<α<1). In addition, assume that there are a large number of consumers in the market, and their judgement of the value of the green agri-food follows a uniform distribution [0,1].

Usually, on PYO farms, consumers can eat part of the green agri-foods they pick and then pay only for the rest. Therefore, the unit price of green agri-foods in PYO channel is often higher than that in online channels. Assume the prices of green agri-foods purchased in PYO channel and online channel are $p_{f}$ and $p_{o}$, satisfying $p_{f}>p_{o}>0$. The unit sale costs of two channels are $c_{f}$ and $c_{o}$, satisfying $v>c_{f}>c_{o}$. Without loss of generality, assume $c_{o}=0$.

In PYO channels, consumers can get the freshest green agri-food. As such, the freshness considered is the initial freshness $\bar{\theta }$. However, when consumers buy green agri-food online, the freshness of the green agri-food $\theta (t_{o})$ attenuates with the passing of time $t_{o}$ for delivery, transportation, and distribution. Assume $\theta (t_{o})=\bar{\theta }-\eta {\left(\frac{t_{o}}{T}\right)}^{2}$, η denotes the freshness decline rate. T is the fresh-keeping period of the green agri-food, and $t_{o}\in [0,T]$.

In the PYO channel, consumers need to bear the transportation cost of commuting between home and the farm, which is denoted as h. Meanwhile, consumers usually do not pay for postage on online channels because of free postage policies. So, the utility functions of consumers in two channels are

(1) $$u_{i}=\{\begin{array}{c} \begin{array}{c} \nu \end{array}-p_{f}+\bar{\theta }-h\begin{array}{c} \left(i=f\right) \end{array}\\ \begin{array}{c} \alpha \nu \end{array}-p_{o}+\theta (t_{o})\begin{array}{c} \left(i=o\right) \end{array} \end{array}$$

The notations used through the paper are summarized in Table 1.

In the equations that follow, the superscript “*” denotes the optimal decision, the superscript “b” denotes the pure PYO mode, the superscript “s” denotes the self-operated dual-channel mode, the superscript “d” denotes the decentralized dual-channel mode, and the superscript “N” denotes the contractual cooperation mode.

We established five models to solve the optimal prices, demands, and profits in different scenarios. The assumptions of five modes are shown in Table 2. 

### 3.2. Benchmark Model (Pure PYO Mode)

When the farmer cooperative only operates the PYO farm, based on the above assumptions, the profit of farmer cooperative is

(2) $$\pi_{f}^{b}(p_{f})=(p_{f}-c_{f})Q_{f}$$

We can obtain that $Q_{f}=1-p_{f}+\theta_{0}-h$ from the consumers utility function. The proof of demand model is shown in Appendix A.

Considering $\frac{\partial^{2}\pi_{f}}{\partial {\left(p_{f}\right)}^{2}}=-2<0$, $\pi_{f}(p_{f})$ is a strictly differentiable concave function. We can solve the first-order condition $\pi_{f}\prime (p_{f})=0$ to obtain the optimal price

(3) $$p_{f}^{b*}=\frac{1}{2}(1+\bar{\theta }+c_{f}-h)$$

The value of optimal price is used to arrive at the demand and profit of the GASC as

(4) $$\{\begin{array}{c} Q^{b*}=\frac{1}{2}(1+\bar{\theta }-h-c_{f})\\ \pi_{f}^{b*}=\frac{1}{4}{\left(1+\bar{\theta }-h-c_{f}\right)}^{2} \end{array}$$

In the pure PYO mode, the offline price is directly proportional to the initial freshness and operation cost, inversely proportional to the transportation cost. The demand is directly proportional to initial freshness, inversely proportional to the operation and transportation cost. The profit is directly proportional to the initial freshness and inversely proportional to the operation and transportation cost. 

## 4. Dual-Channel Models

When $\bar{\theta }-h+\frac{p_{o}T^{2}-\bar{\theta }T^{2}+\eta t_{o}{}^{2}}{\alpha T^{2}}\leq p_{f}\leq 1-\alpha -h+p_{o}+\frac{\eta t_{o}{}^{2}}{T^{2}}$, online and offline channels will coexist in the market at the same time, and the demand functions of two channels are

(5) $$Q_{i}=\{\begin{array}{cc} 1-\frac{p_{f}-p_{o}-\frac{\eta t_{o}{}^{2}}{T^{2}}+h}{1-\alpha } & \left(i=f\right)\\ \frac{p_{f}-p_{o}-\frac{\eta t_{o}{}^{2}}{T^{2}}+h}{1-\alpha }-\frac{p_{o}-\bar{\theta }+\frac{\eta t_{o}{}^{2}}{T^{2}}}{\alpha } & \left(i=o\right) \end{array}$$

The proof of demand functions is shown in Appendix A. 

### 4.1. Self-Operated Dual-Channel Mode

When the farmer cooperative introduced an online channel and operated on its own, it had to pay a fixed fee F to create an online channel. Based on the above assumptions, the profit of farmer cooperative is

(6) $$\pi_{{}^{f}}^{s}(p_{f},p_{o})=(p_{f}-c_{f})Q_{f}+p_{o}Q_{o}-F$$

**Proposition** **1.** *Under the condition L1, the optimal pricing decisions of self-operated dual-channel mode are*

(7) $$\{\begin{array}{c} p_{f}^{s*}=\frac{1}{2}(1+\bar{\theta }+c_{f}-h)\\ p_{o}^{s*}=\frac{1}{2}(\alpha +\bar{\theta }-\frac{\eta t_{o}{}^{2}}{T^{2}}) \end{array}$$

*The value of L1 is shown in the Appendix A.*

*From this, the demands and profits can be obtained as*

(8) $$\{\begin{array}{c} Q_{f}^{s*}=\frac{1}{2}\frac{(1-\alpha -h-c_{f})T^{2}+\eta t_{o}{}^{2}}{(1-\alpha )T^{2}}\\ Q_{o}^{s*}=\frac{1}{2}\frac{(h+c_{f}-\bar{\theta })\alpha T^{2}+\bar{\theta }T^{2}-\eta t_{o}{}^{2}}{(1-\alpha )\alpha T^{2}}\\ Q_{}^{s*}=\frac{1}{2}\frac{(\alpha +\bar{\theta })T^{2}-\eta t_{o}{}^{2}}{\alpha T^{2}}\\ \begin{array}{l} \pi_{f}^{s*}=\frac{1}{4}[(1-2h-2c_{f}+2\bar{\theta })+\frac{(\bar{\theta }-2\eta t_{o}{}^{2})\bar{\theta }}{\alpha }+\frac{{\left(h+c_{f}\right)}^{2}}{1-\alpha }\\ \begin{array}{l} \begin{array}{l} \;-\frac{(h+c_{f})2\eta t_{o}{}^{2}}{(1-\alpha )T^{2}}+\frac{\eta^{2}t_{o}{}^{4}}{(1-\alpha )\alpha T^{4}}]-M \end{array} \end{array} \end{array} \end{array}$$

*In the self-operated dual-channel mode, the offline price is directly proportional to the initial freshness and operation cost, and inversely proportional to the transportation cost. The online price is directly proportional to the initial freshness, brand effect of online channel, and fresh-keeping period, and inversely proportional to the decline rate and delivery time. The demand of the offline channel is directly proportional to the decline rate and delivery time but inversely proportional to the operating cost, transportation cost, and fresh-keeping period, while being unrelated to initial freshness. When* $\bar{\theta }-\theta (t_{o})>h+c_{f}$ *, the demand of the offline channel is directly proportional to the brand effect of online channel. When* $\bar{\theta }-\theta (t_{o})<h+c_{f}$ *, the demand of the offline channel is inversely proportional to the brand effect of online channel. In addition, the demand of the online channel is directly proportional to the operating cost, transportation cost, fresh-keeping period, and initial freshness, while being inversely proportional to the decline rate and delivery time. The total demand is directly proportional to the fresh-keeping period and initial freshness, and inversely proportional to the decline rate, delivery time, and brand effect of online channel, with no correlation with the operating cost and transportation cost. The profit is directly proportional to the initial freshness.*

### 4.2. Decentralized Dual-Channel Mode

When the farmer cooperative collaborates with an online retailer to sell agri-food through an online channel, there are a farmer cooperative and an online retailer in the two-echelon GASC. They make decisions with the goal of maximizing their own profits. In this case, the dual-channel GASC is decentralized. The farmer cooperative and online retailer engage in a Stackelberg game process. First the farmer cooperative decides the wholesale price w and offline price of agri-food $p_{f}$, then the online retailer decides the online price $p_{o}$.

Based on the above assumptions, the profit functions of online retailer, famer cooperative, and the GASC are

(9) $$\pi_{f}^{d}(w,p_{f})=(p_{f}-c_{f})Q_{f}+wQ_{o}$$

(10) $$\pi_{o}^{d}(p_{o})=(p_{o}-w)Q_{o}$$

(11) $$\pi_{}^{d}(p_{f},p_{o})=(p_{f}-c_{f})Q_{f}+p_{o}Q_{o}$$

As the farmer cooperative and online retailer make sequential decisions with the goal of maximizing their own profits, the decentralized dual-channel GASC model which is a bilevel programming problem can be stated as

(12) $$\{\begin{array}{c} \left(p_{f}^{d},w^{d})\in \arg\max_{}\pi_{f}(p_{f},w,p_{o}^{d}(p_{f},w)\right)\\ s.t.p_{o}^{d}(p_{f},w)\in \arg\max\pi_{o}(p_{o}) \end{array}$$

**Proposition** **2.** *Under the condition L2, the optimal pricing decisions of decentralized dual-channel GASC model are*

(13) $$\{\begin{array}{c} p_{f}^{d*}=\frac{1}{2}(1+\bar{\theta }+c_{f}-h)\\ p_{o}^{d*}=\frac{1}{4}[\alpha (2+h+c_{f}-\bar{\theta })+3\bar{\theta }-\frac{3\eta t_{o}{}^{2}}{T^{2}}]\\ w^{d*}=\frac{1}{2}(\alpha +\bar{\theta }-\frac{\eta t_{o}{}^{2}}{T^{2}}) \end{array}$$

*The value of L2 is shown in the Appendix A.*

*From this, the demands and profits can be derived as*

(14) $$\{\begin{array}{c} Q_{f}^{d*}=\frac{1}{4}[h+c_{f}-\bar{\theta }-2-\frac{h+c_{f}}{1-\alpha }-\frac{\eta t_{o}{}^{2}}{(1-\alpha )T^{2}}]\\ Q_{o}^{d*}=\frac{1}{4}[\frac{\bar{\theta }}{\alpha }+\frac{h+c_{f}}{(1-\alpha )\alpha }-\frac{\eta t_{o}{}^{2}}{(1-\alpha )\alpha T^{2}}]\\ \begin{array}{l} Q_{}^{d*}=\frac{1}{4}\frac{[(2-h-c_{f}+\bar{\theta })\alpha +\bar{\theta }]T^{2}-\eta t_{o}{}^{2}}{\alpha T^{2}}\\ \begin{array}{l} \pi_{f}^{d*}=\frac{1}{16} \end{array}\frac{{\left[(h+c_{f}-\bar{\theta })\alpha T^{2}+\bar{\theta }T^{2}-\eta t_{o}{}^{2}\right]}^{2}}{(1-\alpha )\alpha T^{4}}\\ \begin{array}{l} \begin{array}{l} \pi_{o}^{d*}=\frac{1}{16} \end{array}[{\left(h+c_{f}-\bar{\theta }\right)}^{2}+4(h-\bar{\theta }-c_{f})+2+\frac{{\left(h+c_{f}\right)}^{2}}{1-\alpha }+\frac{{\bar{\theta }}^{2}}{\alpha }+\frac{\eta^{2}t_{o}{}^{4}-2\eta t_{o}{}^{2}\bar{\theta }T^{2}}{(1-\alpha )\alpha T^{4}}] \end{array} \end{array}\\ \begin{array}{l} \pi^{d*}=\frac{1}{16}\begin{array}{l} \left[{\left(h+c_{f}-\bar{\theta }\right)}^{2}+4(h-\bar{\theta }-c_{f})+2+\frac{{\left(h+c_{f}\right)}^{2}}{1-\alpha }+\frac{{\bar{\theta }}^{2}}{\alpha }\right] \end{array}\\ +\frac{2\eta^{2}t_{o}{}^{4}+{\left((h+c_{f}-\bar{\theta })\alpha +\bar{\theta }\right)}^{2}T^{4}-2((h+c_{f}-\bar{\theta })\alpha +2\bar{\theta })\eta t_{o}{}^{2}T^{2}}{(1-\alpha )\alpha T^{4}} \end{array} \end{array}$$

In the decentralized dual-channel mode, the offline price is directly proportional to the initial freshness and operation cost, and inversely proportional to transportation cost. However, the online price and the wholesale price are directly proportional to the initial freshness, brand effect of online channel, and fresh-keeping period, while inversely proportional to the decline rate and delivery time. The demand of the offline channel is directly proportional to the decline rate and delivery time, while being inversely proportional to the operating cost, transportation cost of the consumers, and fresh-keeping period, while being unrelated to initial freshness. When $\bar{\theta }-\theta (t_{o})>h+c_{f}$, the demand of the offline channel in the decentralized dual-channel mode is directly proportional to the brand effect of online channel. While $\bar{\theta }-\theta (t_{o})<h+c_{f}$, the demand of the offline channel in the decentralized dual-channel mode is inversely proportional to the brand effect of online channel. The demand of the online channel is directly proportional to the operating cost, transportation cost of the consumers, fresh-keeping period, and initial freshness, and inversely proportional to the decline rate and delivery time. The total demand, while being proportional to the fresh-keeping period and initial freshness, is inversely proportional to the decline rate, delivery time, and brand effect of online channel, and unrelated to the operating cost and transportation cost. The profit of the online retailer is directly proportional to the initial freshness, transportation cost, operating cost, and fresh-keeping period, and inversely proportional to the decline rate and delivery time. The profits are directly proportional to the initial freshness. Moreover, the profit of the farmer cooperative and the GASC is directly proportional to the fresh-keeping period and inversely proportional to the decline rate and delivery time. 

From the above text, we can know that the decentralized dual-channel mode is one of options. However, decentralized supply chain decision-making model will cause double marginalization effect [43,47,54], and the profit of decentralized supply chain is less than that of the centralized supply chain. Therefore, we compare it with the decision-making of centralized dual-channel supply chain which has no marginalization effect but is difficult to achieve in practice, and design a cooperative contract between the farmer cooperatives and the online retailer.

### 4.3. Centralized Dual-Channel Mode

Assume that the farmer cooperative and the online retailer make decisions together with the goal of maximizing the profit of the supply chain, the profit of GASC is

(15) $$\pi (p_{f},p_{o})=(p_{f}-c_{f})Q_{f}+p_{o}Q_{o}$$

**Proposition** **3.** *Under the condition L1, the optimal pricing decisions are*

(16) $$\{\begin{array}{c} p_{f}^{*}=\frac{1}{2}(1+\bar{\theta }+c_{f}-h)\\ p_{o}^{*}=\frac{1}{2}(\alpha +\bar{\theta }-\frac{\eta t_{o}{}^{2}}{T^{2}}) \end{array}$$

*From this, the demand and profit of the GASC can be obtained as*

(17) $$\{\begin{array}{c} Q_{f}^{*}=\frac{1}{2}\frac{(1-\alpha -h-c_{f})T^{2}+\eta t_{o}{}^{2}}{(1-\alpha )T^{2}}\\ Q_{o}^{*}=\frac{1}{2}\frac{(h+c_{f}-\bar{\theta })\alpha T^{2}+\bar{\theta }T^{2}-\eta t_{o}{}^{2}}{(1-\alpha )\alpha T^{2}}\\ Q_{}^{*}=\frac{1}{2}\frac{(\alpha +\bar{\theta })T^{2}-\eta t_{o}{}^{2}}{\alpha T^{2}}\\ \begin{array}{l} \pi^{*}=\frac{1}{4}[(1-2h-2c_{f}+2\bar{\theta })+\frac{(\bar{\theta }-2\eta t_{o}{}^{2})\bar{\theta }}{\alpha }+\frac{{\left(h+c_{f}\right)}^{2}}{1-\alpha }\\ \;-\frac{(h+c_{f})2\eta t_{o}{}^{2}}{(1-\alpha )T^{2}}+\frac{\eta^{2}t_{o}{}^{4}}{(1-\alpha )\alpha T^{4}}] \end{array} \end{array}$$

*From Equations (14) and (17), we know*$\pi^{*}-\pi^{d*}=\frac{{\left[(h+c_{f}-\bar{\theta })\alpha +\theta (t_{o})\right]}^{2}}{16T^{2}(1-\alpha )\alpha }>0$.

*It is inferred that the total profit of the decentralized dual-channel mode is less than centralized dual-channel mode, so the double marginalization effect exists in the decentralized dual-channel mode.*

### 4.4. Contractual Cooperation Mode

The revenue sharing contract is designed to coordinate the decentralized dual-channel supply chain. The online retailer pays the per-unit cost w to the farmer cooperative. At the same time, the farmer cooperative shares a part of the profit with the online retailer. Assume the share is λ, so the share of profit is calculated as $\lambda p_{o}Q_{o}$ and the revenue sharing contract is denoted as (λ,w). The farmer cooperative and the online retailer engage in a three-step process. First, wholesale price w and profit share ratio λ in the revenue sharing contract are decided by the farmer cooperative. Then, the online retailer decides the online price $p_{o}$ and finally, the farmer cooperative decides the offline price $p_{f}$.

The profit functions of online retailer, famer cooperative, and the GASC are

(18) $$\pi_{f}(p_{f})=[(1-\lambda )p_{f}-c_{f}]Q_{f}+wQ_{o}$$

(19) $$\pi_{o}(p_{o})=(p_{o}-w)Q_{o}+\lambda p_{f}Q_{f}$$

(20) $$\pi (p_{f},p_{o})=(p_{f}-c_{f})Q_{f}+p_{o}Q_{o}$$

As the farmer cooperative and the online retailer make sequential decisions with the goal of maximizing their own profits in the decentralized dual-channel supply chain system under the contract, the Stackelberg game model is

(21) $$\{\begin{array}{c} \left(p_{f}^{N})\in \arg\max_{}\pi_{f}(p_{f},\lambda ,w,p_{o}^{N}(p_{f})\right)\\ \begin{array}{ccc} & s.t.p_{o}^{N}(p_{f})\in \arg\max_{}\pi_{o}(p_{o},\lambda ,w) & \end{array} \end{array}$$

$\frac{\partial^{2}\pi_{o}}{\partial {\left(p_{o}\right)}^{2}}=-\frac{2}{1-\alpha }-\frac{2}{\alpha }$, so $\pi_{o}(p_{o})$ is a strictly differentiable concave function. Solving the first-order condition $\pi_{f}^{\prime }(p_{f})=0$, the optimal decision of pricing is obtained as

(22) $$p_{o}^{N*}\left(\lambda ,w,p_{f}\right)=\frac{1}{2}\left(\alpha \lambda p_{f}+\alpha h+\alpha p_{f}-\alpha \bar{\theta }+w+\bar{\theta }-\frac{\eta {t_{o}}^{2}}{T^{2}}\right)$$

Substituting (22) in the profit function of farmer cooperatives (18), $\pi_{f}(p_{f},\lambda ,w,p_{o}^{N}(p_{f},\lambda ,w))$ is obtained.

Since $\frac{\partial^{2}\pi_{f}}{\partial {\left(p_{f}\right)}^{2}}=-\frac{\left(1-\lambda \right)\left(2-\alpha \lambda -\alpha \right)}{1-\alpha }<0$, $\pi_{f}(p_{f})$ is a strictly differentiable concave function. Solving the first-order condition $\pi_{f}^{\prime }(p_{f})=0$, the optimal decision of pricing is

(23) $$p_{f}^{N*}\left(\lambda ,w\right)=\frac{\alpha \bar{\theta }-\alpha h+2h+2\alpha -\bar{\theta }-2w}{2\left(\alpha \lambda +\alpha -2\right)}+\frac{h-1}{\left(1-\lambda \right)\left(\alpha \lambda +\alpha -2\right)}-\frac{\eta {t_{o}}^{2}}{2T^{2}\left(\alpha \lambda +\alpha -2\right)}+\frac{c_{f}}{2\left(1-\lambda \right)}$$

Substituting (23) in the online price function (22), we get

(24) $$p_{o}^{N*}\left(\lambda ,w\right)=\frac{[\left(2h+\bar{\theta }-2\right)\lambda^{2}+\left(2c_{f}-4h+4\bar{\theta }\right)\lambda +2h+2c_{f}-5\bar{\theta }+2]\alpha }{4\left(1-\lambda \right)\left[2-\left(\lambda +1\right)\alpha \right]}+\frac{w+\bar{\theta }}{2-(\lambda +1)\alpha }-\frac{\eta {t_{o}}^{2}}{2T^{2}}$$

To eliminate the double marginalization effect of decentralized decision making, the total profit of the decentralized dual-channel supply chain should be equal to the total profit of the centralized dual-channel supply chain. The equilibrium result of the decentralized dual-channel supply chain should be equal to the equilibrium result of the centralized dual-channel supply chain.

(25) $$\{\begin{array}{c} p_{f}^{N*}=p_{f}^{*}\\ p_{o}^{N*}=p_{o}^{*} \end{array}$$

Thus, Equations (19), (23), (24) and (25) are solved to obtained the revenue sharing contract parameters for perfect coordination.

(26) $$\left\{\begin{array}{c} \lambda^{*}=1-\frac{2\left(1-\alpha \right)c_{f}}{\alpha h-\alpha c_{f}-\alpha \bar{\theta }+2c_{f}+\theta \left(t_{o}\right)}\\ w^{N*}=\frac{\left(1-\alpha -h-c_{f}+\eta \frac{{t_{o}}^{2}}{T^{2}}\right)\alpha c_{f}}{\alpha h-\alpha c_{f}-\alpha \bar{\theta }+2c_{f}+\theta \left(t_{o}\right)} \end{array}\right.$$

The specific profit function of the online retailer and farmer cooperative under the revenue sharing contract is not presented due to its complexity; instead, the numerical analysis is outlined in the Section 6.

After adopting the revenue sharing contract (λ,w), the online retailer earns more profit than before, but the farmer cooperative’s profit is less than before. Therefore, the online retailer has enough incentive to adopt this contract, while the farmer cooperative does not. 

To give both the online retailer and the farmer cooperative the incentive to adopt the contract and coordinate, it is necessary to include a fixed transfer payment M in the revenue sharing contract. This means that the revenue sharing and transfer payment contract (λ,w,M) need to meet the conditions of

(27) $$\{\begin{array}{c} \pi_{f}^{N*}=\pi_{f}^{N}+M>\pi_{f}^{d*}\\ \pi_{o}^{N*}=\pi_{o}^{N}-M>\pi_{o}^{d*} \end{array}$$

The range of the fixed transfer payment is computed as

(28) $$M\in [\frac{\left[\left(E-B+h^{2}\right)\alpha -\left(2h\bar{\theta }-B\right)\right]\alpha AT^{4}-DA}{8\alpha T^{4}\left(A+2c_{f}T^{2}\right)\left(1-\alpha \right)},\frac{\left[\left(E-2B+3h^{2}\right)\alpha -\left(2{\bar{\theta }}^{2}-2B+2h\bar{\theta }-2{c_{f}}^{2}\right)\right]\alpha AT^{4}-3DA}{16\alpha T^{4}\left(A+2c_{f}T^{2}\right)\left(1-\alpha \right)}]$$

The values of A, B, D, and E are shown in Appendix A.

## 5. Model Comparison and Analysis

We obtain the following corollaries by comparing the optimal solutions of five different scenarios, and the proofs of corollaries are shown in Appendix A.

**Corollary** **1.** *The optimal cooperative’s profit is model in four modes, except for centralized dual-channel mode. In this mode, supply chain members aim at maximizing the total profit of the supply chain, so it is impossible to point out the individual profit of the farmer cooperative. The optimal farmer cooperative’s profit of the four modes in descending order are:*

(29) $$\{\begin{array}{ll} \pi_{f}^{s*}>\pi_{f}^{N*}>\pi_{f}^{d*}>\pi_{f}^{b*} & 0<F<\frac{2}{16}G\begin{array}{l} \begin{array}{l} and\begin{array}{l} \pi_{f}^{N}+M_{\max}<\pi_{f}^{s*} \end{array} \end{array} \end{array}\\ \pi_{f}^{N*}>\pi_{f}^{s*}>\pi_{f}^{d*}>\pi_{f}^{b*} & 0<F<\frac{2}{16}G\begin{array}{l} \begin{array}{l} and\begin{array}{l} \pi_{f}^{N}+M_{\max}>\pi_{f}^{s*} \end{array} \end{array} \end{array}\\ \pi_{f}^{N*}>\pi_{f}^{d*}>\pi_{f}^{s*}>\pi_{f}^{b*} & \frac{2}{16}G<F<\frac{4}{16}G\\ \pi_{f}^{N*}>\pi_{f}^{d*}>\pi_{f}^{b*}>\pi_{f}^{s*} & \frac{4}{16}G<F \end{array}$$

*When the fixed cost of creating a new online channel*$F<\frac{2}{16}G$ and $\pi_{f}^{N}+M_{\max}<\pi_{f}^{s*}$, the profit of the farmer cooperative in self-operated dual-channel mode is the highest. In other cases, the profit of the farmer cooperative in contractual cooperation mode is the highest. The profit of the farmer cooperative in decentralized dual-channel mode is always more than the profit of the farmer cooperative in pure PYO mode. When the fixed cost of creating a new online channel $F>\frac{4}{16}G$*, the profit of the farmer cooperative in self-operated dual-channel mode is less than the pure PYO mode.*

**Corollary** **2.** *The optimal GASC’s profit of the five modes in descending order are*

(30) $$\{\begin{array}{ll} \pi^{N*}=\pi^{*}>\pi_{}^{s*}>\pi^{d*}>\pi_{}^{b*} & 0<F<\frac{1}{16}G\\ \pi^{N*}=\pi^{*}>\pi^{d*}>\pi_{}^{s*}>\pi_{}^{b*} & \frac{1}{16}G<F<\frac{4}{16}G\\ \pi^{N*}=\pi^{*}>\pi^{d*}>\pi_{}^{b*}>\pi_{}^{s*} & \frac{4}{16}G<F \end{array}$$

*The profit of GASC in contractual cooperation mode and centralized dual-channel mode are always the highest. The profit of GASC in decentralized dual-channel mode is always more than the profit of GASC in pure PYO mode. When the fixed cost of creating a new online channel* $F>\frac{4}{16}G$ *, the profit of GASC in self-operated dual-channel mode is less than the pure PYO mode. The value of* G *is shown in Appendix A.*

**Corollary** **3.** *The optimal demands of the five modes in descending order are*

*(I)* $Q_{o}^{s*}=Q_{o}^{*}=Q_{o}^{N*}>Q_{o}^{d*}$

*(II) when* $\bar{\theta }>h+c_{f}$ *,* $Q^{s*}=Q^{*}=Q^{N*}>Q_{}^{d*}>Q^{b*}>Q_{f}^{d*}>Q_{f}^{s*}=Q_{f}^{*}=Q_{f}^{N*}$ *;*

(31) $$\begin{array}{c} \mathrm{when}\;\bar{\theta }<h+c_{f},\;\\ \{\begin{array}{c} Q_{}^{d*}>Q^{b*}>Q^{s*}=Q^{*}=Q^{N*}>Q_{f}^{d*}>Q_{f}^{s*}=Q_{f}^{*}=Q_{f}^{N*}\begin{array}{cc} & \alpha >\frac{\theta (t_{o})}{h+c_{f}-\bar{\theta }} \end{array}\\ \begin{array}{cc} Q^{s*}=Q^{*}=Q^{N*}>Q_{}^{d*}>Q^{b*}>Q_{f}^{d*}>Q_{f}^{s*}=Q_{f}^{*}=Q_{f}^{N*} & \alpha <\frac{\theta (t_{o})}{h+c_{f}-\bar{\theta }} \end{array} \end{array}. \end{array}$$

*The demand of the online channel in the decentralized dual-channel mode is less than that in the self-operated dual-channel mode, contractual cooperation mode and centralized dual-channel mode, while the demand of the offline channel in the decentralized dual-channel mode is always more than that in the self-operated dual-channel mode, contractual cooperation mode, and centralized dual-channel mode. The relationship among the total demands of the GASC is related to freshness, transportation cost, offline marketing cost, and online brand effect.*

**Corollary** **4.** *The relationships among the optimal prices of the five modes are*

*(I)* $p_{f}^{b*}=p_{f}^{s*}=p_{f}^{d*}=p_{f}^{*}=p_{f}^{N*}$

*(II)* $p_{o}^{d*}>p_{o}^{s*}=p_{o}^{*}=p_{o}^{N*}=w_{}^{d*}$

*The offline prices in five modes are the same. The wholesale price in the decentralized dual-channel mode is less than the online price in the decentralized dual-channel mode, and is equal to the online price in self-operated dual-channel mode, contractual cooperation mode, and decentralized dual-channel mode.*

The optimal decisions of five modes are shown in Table 3.

## 6. Numerical Experimentation

According to the assumptions and constraints in the model, the specific values of the parameters are set at α=0.7, h=0.01, $c_{f}=0.04$, $\bar{\theta }=0.6$, $t_{o}=0.02$, T=0.03, η=0.02, F=0.06. The results are detailed in Table 4.

In this numerical example, we have $F>\frac{2}{16}G$. It can be verified from Table 4 that the total profits in centralized dual-channel mode and contractual cooperation mode are the highest, followed by the decentralized dual-channel mode, then the pure PYO mode, and finally by the self-operated dual-channel mode. The profit of the farmer cooperative in contractual cooperation mode is the highest. The total demands in self-operated dual-channel mode, centralized dual-channel mode, and contractual cooperation mode are the highest, followed by the decentralized dual-channel mode, and finally by the pure PYO mode. The offline prices are the same in all the five cases. The online price in decentralized dual-channel mode is higher than that in the self-operated dual-channel mode, contractual cooperation mode, and centralized dual-channel mode, while the online demand in decentralized dual-channel mode is lower than that in the other three modes. Next, we discuss the impact of the brand effect of the online channel and initial freshness on decision-making.

### 6.1. Brand Effect of Online Channel

**Figure 2:**
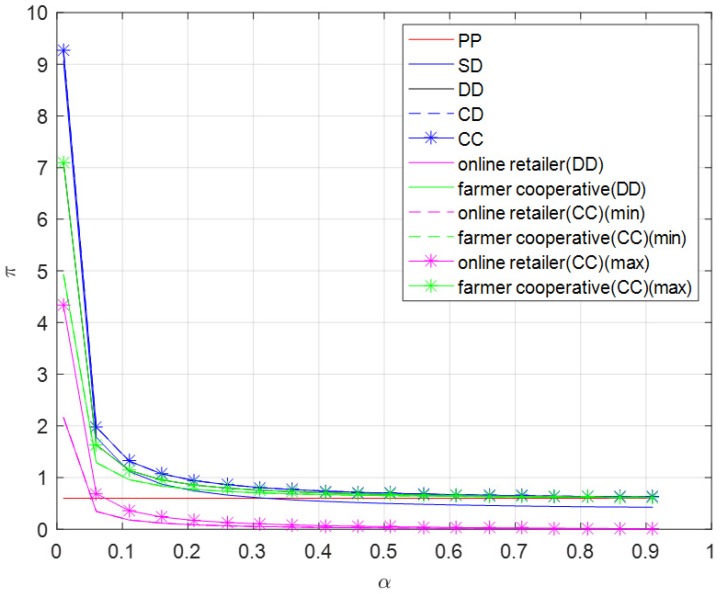
Optimal profit versus α.

It can be observed from Figure 2 that the profit of pure PYO mode has nothing to do with brand effect of online channel, while other profits decrease with the increase of brand effect of the online channel. When the brand effect of the online channel is relatively small, profits other than the profit of pure PYO mode decline rapidly. When the brand effect of online channel is relatively large, profits other than the profit of pure PYO mode change more moderately. The total profits in centralized dual-channel mode and contractual cooperation mode are always the largest. When the brand effect of online channel is small, the self-operated dual-channel mode’s profit is the third largest, and more than the total profit in pure PYO mode and decentralized dual-channel mode. When the brand effect of the online channel is relatively large, the self-operated dual-channel mode’s profit is the fourth smallest, less than the total profit of the other four modes. 

**Figure 3:**
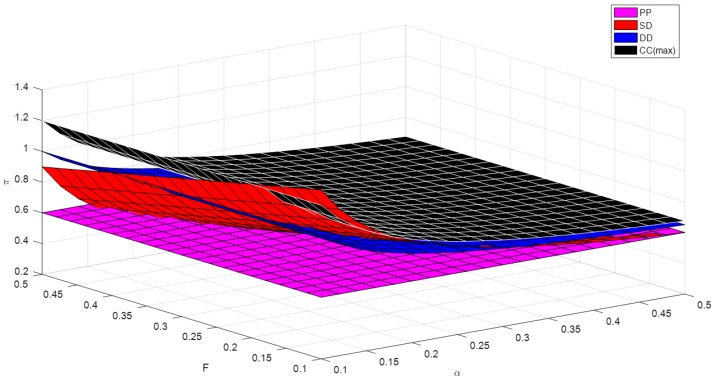
Optimal profit of the farmer cooperative versus α and F.

As shown in Figure 3, when the brand effect of online channel and the fixed cost of creating a new online channel are relatively small, the optimal profit of the farmer cooperative in self-operated dual-channel mode is the largest, followed by the contractual cooperation, then the decentralized dual-channel mode, finally the pure PYO mode. when the brand effect of online channel is relatively small, and the fixed cost of creating a new online channel is a little larger, the optimal profit of the farmer cooperative in contractual cooperation mode is the largest, followed by the self-operated dual-channel mode, then the decentralized dual-channel mode, finally the pure PYO mode. In most of the remaining areas, the optimal profit of the farmer cooperative in contractual cooperation is the largest, followed by the decentralized dual-channel mode, then the self-operated dual-channel mode, finally the pure PYO mode.

**Figure 4:**
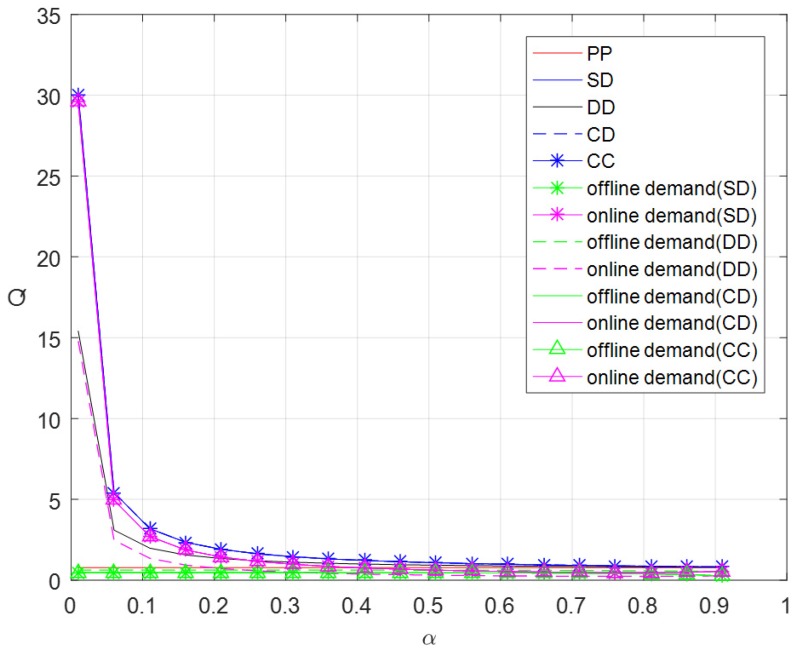
Optimal demand versus α.

From Figure 4, the demand of pure PYO mode has nothing to do with brand effect of online channel, while other demands decrease with the increase of brand effect of online channel. When the brand effect of online channel is relatively small, demands other than the pure PYO mode decline rapidly. When the brand effect of the online channel is relatively large, demands other than the pure PYO mode change more moderately. The total demands in self-operated dual-channel mode, centralized dual-channel mode and contractual cooperation mode are always the largest. When the brand effect of the online channel is relatively small, the online demand becomes greater than offline demands. With the increase of brand effect of the online channel, the relationship between demands will change. It can be concluded that the demand of the online channel will decrease, and will be smaller than that of the offline channel. At the same time, the total demand of the dual channel model will also decrease, and finally come close to that of the single channel.

**Figure 5:**
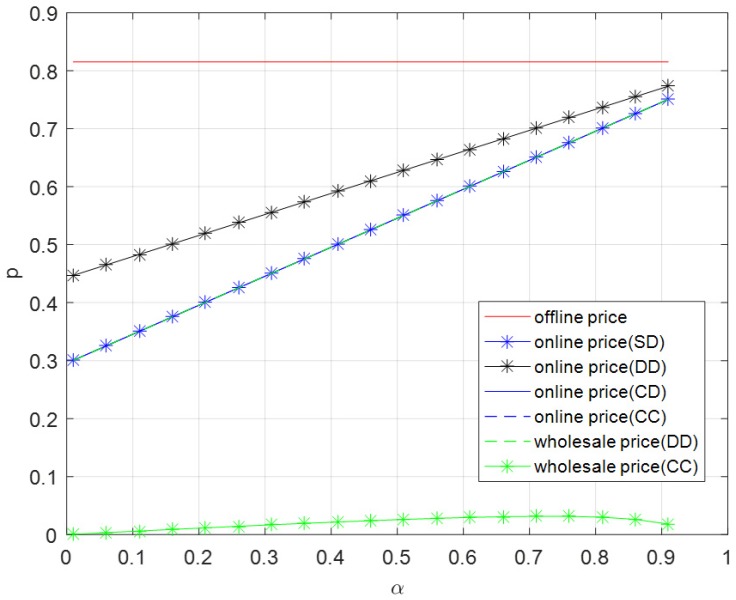
Optimal price versus α.

As shown in Figure 5, offline prices do not change with the brand effect of online channel, and they are equal in five modes. Online prices and the wholesale price in the decentralized dual-channel mode increase with the brand effect of online channel. The highest price is the offline price, followed by the online price in decentralized dual-channel mode, then online prices in three other modes and the wholesale price in decentralized dual-channel mode, and finally the wholesale price in contractual cooperation mode. The difference value between prices (except the wholesale price in the contractual cooperation mode) decreases as the brand effect of online channel increases.

### 6.2. Initial Freshness 

**Figure 6:**
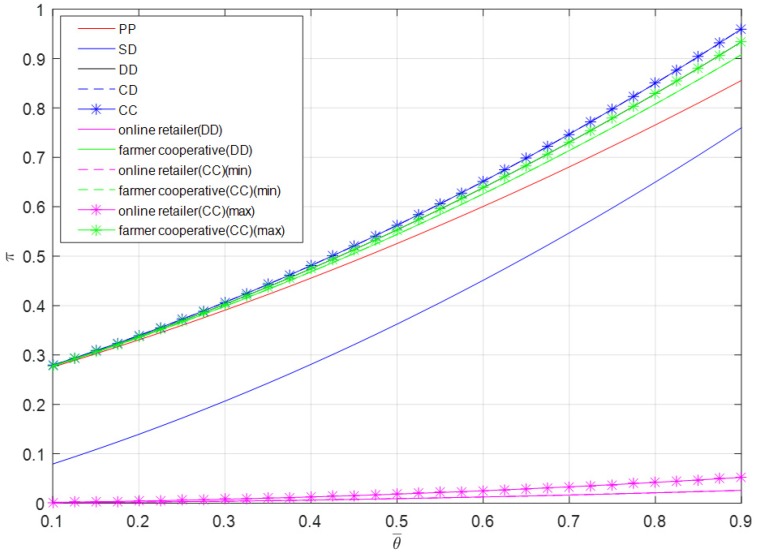
Optimal profit versus $\bar{\theta }$.

It can be observed from Figure 6 that all stakeholders’ profits increase with an increase in initial freshness. The change of initial freshness has more significant impact on the profit of the offline channel. The total profits in centralized dual-channel mode and contractual cooperation mode are the largest, followed by the decentralized dual-channel mode’s total profit, the profits of the farmer cooperative, the pure PYO mode’s total profit, the self-operated dual-channel mode’s total profit, and the online retailer’s profits.

**Figure 7:**
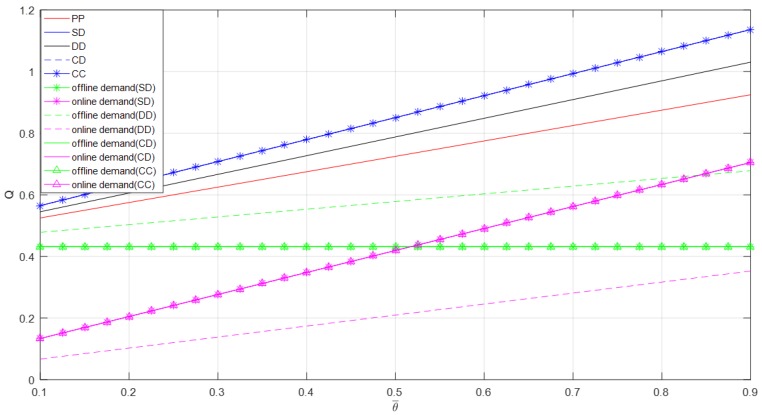
Optimal demand versus $\bar{\theta }$.

As shown in Figure 7, the demands increase with an increase in initial freshness. The total demands in self-operated dual-channel mode, centralized dual-channel mode and contractual cooperation mode are the largest, followed by decentralized dual-channel mode’s total demand, and pure PYO mode’s total demand. The online demand of the decentralized dual-channel mode is less than the online demands of self-operated dual-channel mode, centralized dual-channel mode, and contractual cooperation mode. While the offline demand of the decentralized dual-channel mode is larger than the demands of other three modes. In the self-operated dual-channel mode, centralized dual-channel mode, and contractual cooperation mode, the online demands are less than the offline demands, but when the initial freshness increases to a certain value, the online demands are larger than the offline demands. Online demand of the decentralized dual-channel mode is less than its offline demand. 

**Figure 8:**
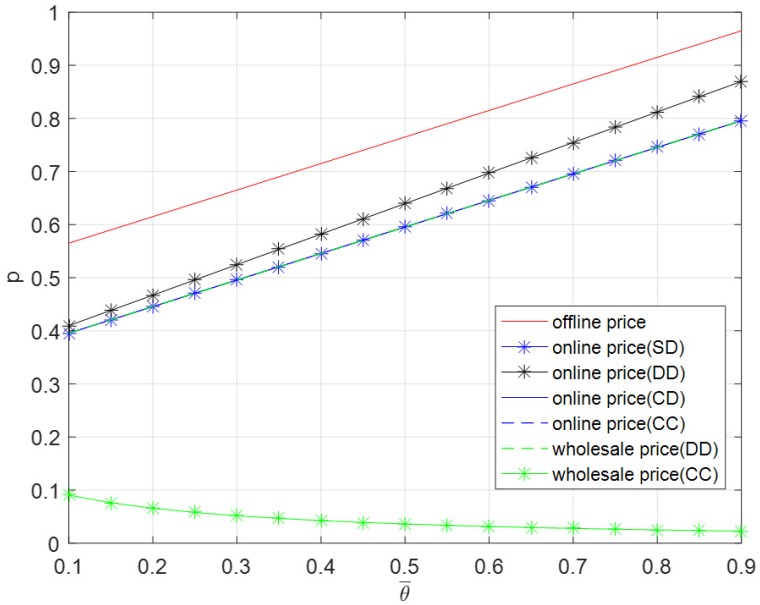
Optimal price versus $\bar{\theta }$.

From Figure 8, as the initial freshness increases, the offline price, online price, and wholesale price in decentralized dual-channel mode increase, while the wholesale price in contractual cooperation mode decreases. The offline prices are equal in five modes, the wholesale price in decentralized dual-channel mode is equal to the online prices in the self-operated dual-channel mode, centralized dual-channel mode, and contractual cooperation mode. The highest price is the offline price, followed by the online price in decentralized dual-channel mode, then the online prices in the other three modes and the wholesale price in decentralized dual-channel mode, and finally the wholesale price in contractual cooperation mode. 

## 7. Discussion

From the perspective of consumer experience, based on the freshness of green agri-food, transportation cost, and other factors, the utility functions of online and offline channels are considered respectively. Then, we derive the demand function from the utility function and establish profit models to investigate the introduction of online channel in PYO operation supply chain. In general, we obtain six findings in this paper.

(1) When the fixed cost of creating a new online channel and brand effect of online channel are relatively small, there are two possibilities. The profit of the farmer cooperative in self-operated dual-channel mode is the highest, or that in contractual cooperation mode is the highest. When the fixed cost is relatively large, the profit of the farmer cooperative in contractual cooperation mode is always the highest. The profit of the farmer cooperative in contractual cooperation mode is always more than that in decentralized dual-channel mode which is always more than the profit of the farmer cooperative in pure PYO mode, that means—from a profit point of view—introducing online channel is always better than not for the farmer cooperative. This is different from literatures [50,51,52,53], in which online channels are introduced only under certain conditions. When the fixed cost increases to a certain threshold, the profit of the farmer cooperative in self-operated dual-channel mode is less than the pure PYO mode, to be the smallest of all modes.

(2) The revenue sharing and fixed transfer payment contract could facilitate coordination in the decentralized dual-channel mode. This is similar to the studies of [20,26,54], which use revenue sharing contracts to coordinate supply chains. The total profit of the GASC in contractual cooperation mode is equal to that in the centralized dual-channel mode, and they are always the largest. The total profit in decentralized dual-channel mode is always more than the total profit in pure PYO mode. As the fixed cost increases, the total profit in self-operated dual-channel mode will change from the second highest to the smallest of all modes. 

(3) The total demands of the GASC the demands of the online channel and the demands of the offline channel in the self-operated dual-channel mode, contractual cooperation mode, and centralized dual-channel mode are equal respectively. The total demand of the GASC in the decentralized dual-channel mode is always more than that in the pure PYO mode. When the initial freshness is relatively small and the brand benefit of online channel is relatively large, the total demand in the decentralized dual-channel mode is the largest, followed by the pure PYO mode, and then the other three modes, while in the remaining cases, the three modes are the largest. The demand of the online channel in the decentralized dual-channel mode is less than that in the self-operated dual-channel mode, contractual cooperation mode, and centralized dual-channel mode, while the demand of the offline channel in the decentralized dual-channel mode is always more than that in other three modes. The demand of offline channel in any mode is always less than the total demand in any mode. 

(4) The offline prices were equal in five modes. The wholesale price in the decentralized dual-channel mode is equal to the online price in self-operated dual-channel mode, contractual cooperation mode, and decentralized dual-channel mode, and less than the online price in the decentralized dual-channel mode.

(5) Offline prices, pure PYO mode’s profit, and pure PYO mode’s total demand are directly proportional to the initial freshness. The online prices, wholesale price, total demands, online demands, profits of the farmer cooperative, and profits of the online retailer are all directly proportional to the initial freshness and the fresh-keeping period, and are inversely proportional to the delivery time and decline rate. The offline demands are directly proportional to the initial freshness, delivery time, and decline rate, and are inversely proportional to the fresh-keeping period. 

(6) The profits of the GASC do not increase monotonously with the increase of brand effect of online channel, even in some cases, they decrease with the increase of brand effect. The profits of the farmer cooperative and the online retailer also decrease with the increase of brand effect. When the brand effect of online channel is very small, the small change of the brand effect of online channel can cause a big change in profits. The demands of the GASC also show similar characteristics, while online prices increase with the brand effect of online channel. The profit-sharing parameter in contracts increases with the brand effect of online channel. After introducing the online channel, when the freshness loss of the online channel is greater than the sum of the transportation and operation costs, the offline demands of dual channel are proportional to the brand effect of the online channel. When the freshness loss of the online channel is less than the sum of the transportation and operation costs, the offline demands of dual channel are inversely proportional to the brand effect of online channel.

## 8. Conclusion

After introducing the online channel to the GASC with PYO operation, we collected and analyzed the results. We studied how the online channel would impact the business and based on parameters like online channel’s brand effect, freshness, we concluded the following management implications.

(1) PYO operation, a sustainable offline marketing, can bring more profits when combined with online marketing. Farmer cooperatives should introduce online channel in the PYO operation GASC. When the cost of creating a new online shopping channel is small, farmer cooperatives can operate online channels by themselves. Meanwhile, they should also pay attention to the brand effect of online channel within a certain range. When the cost of creating a new online shopping channel is large, they can choose to cooperate with online retailers. In addition, it would grant GASC more profit when they could adopt contracts to govern cooperation with online retailers which makes it a win-win for both sides.

(2) When an online channel exists, regardless of who is running it, the brand effect of online channel is simply not the stronger the better. If the brand of the online channel is too strong, it may compete fiercely with the offline channel, resulting in a drop of demand for both online and offline channels, thus lowering profits.

(3) Introducing online channels will divert the demand of offline channels. For some PYO farms with less offline demand or in more remote locations, more consideration should be given to introducing online channels. Efforts should be made to reduce operating costs. When operating costs and transportation costs are relatively large, more attention should be paid to controlling the development of online channel brand’s effect, otherwise competition will intensify and total demand will be reduced.

(4) Providing fresh green agri-foods is good for consumers’ health. At the same time, it can also bring more profits to farmer cooperatives and online retailers. In order to increase total sales, it is necessary to pick agri-foods with an appropriate level of freshness instead of waiting for an offline customer to pick them. Green agri-foods with a longer fresh-keeping period, that is, less perishable agri-foods are more viable options for online channel sales.

In order to simplify the calculation, some assumptions and constraints are made in this paper. This limits this paper in some extent, but they are also extensible directions for future research. Further research can look into the distribution model between the provider and the consumer when the distance between them might alter the results. Secondly, the consumer preference between channels varies by different regions and ages, where we were limited by the same preference. Thirdly, consumers eat and possibly waste some green agri-food during picking. These should also be taken into account. Finally, green agri-foods are perishable and may not be a constant price during the shelf life. Stage pricing strategy may be closer to reality. 

## Figures and Tables

**Table 1:** Notations.

Symbols	Descriptions
f and o	PYO channel and online channel
$\nu_{i}$	Consumers’ value assessments for green agri-good in channel *i*, assume $v_{f}=v>v_{o}=\alpha v>0$
$p_{i}$	Price of green agri-food purchased in channel *i*, assume $p_{f}>p_{o}>0$
$c_{i}$	Unit sale cost of channel *i*, assume $c_{f}>c_{o}=0$
$Q_{i}$	Demand in channel *i*
$\bar{\theta }$	Initial freshness
$\theta (t_{o})$	Freshness of green agri-food purchased in online channel
η	Freshness decline rate
T	Fresh-keeping period of green agri-food
$t_{o}$	Time from picking to customer’s hands via an online channel
h	Transportation cost of commuting between home and farm

**Table 2:** Assumptions of five modes.

Mode	Members	Cooperation	Goal
Pure PYO mode (PP)	The farmer cooperative	-	Maximizing its own profit
Self-operated dual-channel mode (SD)			
Centralized dual-channel mode (CD)	The farmer cooperative, the online retailer	Make decisions together with same goal	Maximizing the overall profit of the GASC
Decentralized dual-channel mode (DD)		Compete against each other with their own goals	Maximizing their own profit respectively
Contractual cooperation mode (CC)		Sharing the revenue together and transferring the payment during decentralized decision-making	

PP, pure PYO mode; SD, self-operated dual-channel mode; DD, decentralized dual-channel mode; CD, centralized dual-channel mode; CC, contractual cooperation mode.

**Table 3:** Optimal decisions of five modes.

Modes	Pure PYO Mode (PP)	Self-Operated Dual-Channel Mode (SD)	Decentralized Dual-Channel Mode (DD)	Centralized Dual-Channel Mode (CD)	Contractual Cooperation Mode (CC)
Offline price	$p_{f}^{b*}=\frac{1}{2}(1+\bar{\theta }+c_{f}-h)$	$p_{f}^{s*}=\frac{1}{2}(1+\bar{\theta }+c_{f}-h)$	$p_{f}^{d*}=\frac{1}{2}(1+\bar{\theta }+c_{f}-h)$	$p_{f}^{*}=\frac{1}{2}(1+\bar{\theta }+c_{f}-h)$	$p_{f}^{N*}=\frac{1}{2}(1+\bar{\theta }+c_{f}-h)$
Online price	-	$p_{o}^{s*}=\frac{1}{2}(\alpha +\bar{\theta }-\frac{\eta t_{o}{}^{2}}{T^{2}})$	$\begin{array}{l} p_{o}^{d*}=\frac{1}{4}[\alpha (2+h+c_{f}-\bar{\theta })\\ +3\bar{\theta }-\frac{3\eta t_{o}{}^{2}}{T^{2}}] \end{array}$	$p_{o}^{*}=\frac{1}{2}(\alpha +\bar{\theta }-\frac{\eta t_{o}{}^{2}}{T^{2}})$	$p_{o}^{N*}=\frac{1}{2}(\alpha +\bar{\theta }-\frac{\eta t_{o}{}^{2}}{T^{2}})$
Wholesale price	-	-	$w^{d*}=\frac{1}{2}(\alpha +\bar{\theta }-\frac{\eta t_{o}{}^{2}}{T^{2}})$	-	
Offline demand	$Q^{b*}=\frac{1}{2}(1+\bar{\theta }-h-c_{f})$	$Q_{f}^{s*}=\frac{1}{2}\frac{(1-\alpha -h-c_{f})T^{2}+\eta t_{o}{}^{2}}{(1-\alpha )T^{2}}$	$\begin{array}{l} Q_{f}^{d*}=\frac{1}{4}[h+c_{f}-\bar{\theta }-2-\frac{h+c_{f}}{1-\alpha }\\ -\frac{\eta t_{o}{}^{2}}{(1-\alpha )T^{2}}] \end{array}$	$Q_{f}^{*}=\frac{1}{2}\frac{(1-\alpha -h-c_{f})T^{2}+\eta t_{o}{}^{2}}{(1-\alpha )T^{2}}$	$Q_{f}^{N*}=\frac{1}{2}\frac{(1-\alpha -h-c_{f})T^{2}+\eta t_{o}{}^{2}}{(1-\alpha )T^{2}}$
Online demand	-	$Q_{o}^{s*}=\frac{1}{2}\frac{(h+c_{f}-\bar{\theta })\alpha T^{2}+\bar{\theta }T^{2}-\eta t_{o}{}^{2}}{(1-\alpha )\alpha T^{2}}$	$Q_{o}^{d*}=\frac{1}{4}[\frac{\bar{\theta }}{\alpha }+\frac{h+c_{f}}{(1-\alpha )\alpha }-\frac{\eta t_{o}{}^{2}}{(1-\alpha )\alpha T^{2}}]$	$Q_{o}^{*}=\frac{1}{2}\frac{(h+c_{f}-\bar{\theta })\alpha T^{2}+\bar{\theta }T^{2}-\eta t_{o}{}^{2}}{(1-\alpha )\alpha T^{2}}$	$Q_{o}^{N*}=\frac{1}{2}\frac{(h+c_{f}-\bar{\theta })\alpha T^{2}+\bar{\theta }T^{2}-\eta t_{o}{}^{2}}{(1-\alpha )\alpha T^{2}}$
Total demand	$Q^{b*}=\frac{1}{2}(1+\bar{\theta }-h-c_{f})$	$Q_{}^{s*}=\frac{1}{2}\frac{(\alpha +\bar{\theta })T^{2}-\eta t_{o}{}^{2}}{\alpha T^{2}}$	$Q_{}^{d*}=\frac{[(2-h-c_{f}+\bar{\theta })\alpha +\bar{\theta }]T^{2}-\eta t_{o}{}^{2}}{4\alpha T^{2}}$	$Q_{}^{*}=\frac{1}{2}\frac{(\alpha +\bar{\theta })T^{2}-\eta t_{o}{}^{2}}{\alpha T^{2}}$	$Q_{}^{N*}=\frac{1}{2}\frac{(\alpha +\bar{\theta })T^{2}-\eta t_{o}{}^{2}}{\alpha T^{2}}$
Farmer cooperative’s profit	$\pi_{f}^{b*}=\frac{1}{4}{\left(1+\bar{\theta }-h-c_{f}\right)}^{2}$	$\begin{array}{l} \pi_{f}^{s*}=\frac{1}{4}[(1-2h-2c_{f}+2\bar{\theta })+\frac{{\left(h+c_{f}\right)}^{2}}{1-\alpha }\\ \begin{array}{l} \begin{array}{l} \begin{array}{l} +\frac{(\bar{\theta }-2\eta t_{o}{}^{2})\bar{\theta }}{\alpha }-\frac{(h+c_{f})2\eta t_{o}{}^{2}}{(1-\alpha )T^{2}}\\ +\frac{\eta^{2}t_{o}{}^{4}}{(1-\alpha )\alpha T^{4}}]-M \end{array} \end{array} \end{array} \end{array}$	$\begin{array}{l} \pi_{f}^{d*}= \end{array}\frac{{\left[(h+c_{f}-\bar{\theta })\alpha T^{2}+\bar{\theta }T^{2}-\eta t_{o}{}^{2}\right]}^{2}}{16(1-\alpha )\alpha T^{4}}$	-	$\pi_{f}^{N*}=\pi_{f}^{N}+M>\pi_{f}^{d*}$
Online retailer’s profit	-	-	$\begin{array}{l} \begin{array}{l} \pi_{o}^{d*}=\frac{1}{16} \end{array}[{\left(h+c_{f}-\bar{\theta }\right)}^{2}+4(h-\bar{\theta }-c_{f})+2\\ +\frac{{\left(h+c_{f}\right)}^{2}}{1-\alpha }+\frac{{\bar{\theta }}^{2}}{\alpha }+\frac{\eta^{2}t_{o}{}^{4}-2\eta t_{o}{}^{2}\bar{\theta }T^{2}}{(1-\alpha )\alpha T^{4}}] \end{array}$	-	$\pi_{o}^{N*}=\pi_{o}^{N}-M>\pi_{o}^{d*}$
Total profit	$\pi_{}^{b*}=\frac{1}{4}{\left(1+\bar{\theta }-h-c_{f}\right)}^{2}$	$\begin{array}{l} \pi_{}^{s*}=\frac{1}{4}[(1-2h-2c_{f}+2\bar{\theta })+\frac{{\left(h+c_{f}\right)}^{2}}{1-\alpha }\\ \begin{array}{l} \begin{array}{l} \begin{array}{l} +\frac{(\bar{\theta }-2\eta t_{o}{}^{2})\bar{\theta }}{\alpha }-\frac{(h+c_{f})2\eta t_{o}{}^{2}}{(1-\alpha )T^{2}}\\ +\frac{\eta^{2}t_{o}{}^{4}}{(1-\alpha )\alpha T^{4}}]-M \end{array} \end{array} \end{array} \end{array}$	$\begin{array}{l} \pi^{d*}=\frac{1}{16}\begin{array}{l} \left[{\left(h+c_{f}-\bar{\theta }\right)}^{2}+4(h-\bar{\theta }-c_{f}\right) \end{array}\\ +\frac{{\left(h+c_{f}\right)}^{2}}{1-\alpha }]+\frac{{\bar{\theta }}^{2}}{16\alpha }+\frac{2\eta^{2}t_{o}{}^{4}}{(1-\alpha )\alpha T^{4}}\\ -\frac{2[(h+c_{f}-\bar{\theta })\alpha +2\bar{\theta }]\eta t_{o}{}^{2}}{(1-\alpha )\alpha T^{2}}\\ +\frac{{\left[(h+c_{f}-\bar{\theta })\alpha +\bar{\theta }\right]}^{2}}{(1-\alpha )\alpha }+\frac{1}{8} \end{array}$	$\begin{array}{l} \pi^{*}=\frac{1}{4}[(1-2h-2c_{f}+2\bar{\theta })+\frac{{\left(h+c_{f}\right)}^{2}}{1-\alpha }\\ -\frac{(h+c_{f})2\eta t_{o}{}^{2}}{(1-\alpha )T^{2}}+\frac{\eta^{2}t_{o}{}^{4}}{(1-\alpha )\alpha T^{4}}\\ +\frac{(\bar{\theta }-2\eta t_{o}{}^{2})\bar{\theta }}{\alpha }] \end{array}$	$\begin{array}{l} \pi^{N*}=\frac{1}{4}[(1-2h-2c_{f}+2\bar{\theta })+\frac{{\left(h+c_{f}\right)}^{2}}{1-\alpha }\\ -\frac{(h+c_{f})2\eta t_{o}{}^{2}}{(1-\alpha )T^{2}}+\frac{\eta^{2}t_{o}{}^{4}}{(1-\alpha )\alpha T^{4}}\\ +\frac{(\bar{\theta }-2\eta t_{o}{}^{2})\bar{\theta }}{\alpha }] \end{array}$

* The specific profit functions of the online retailer and farmer cooperative under the revenue sharing contract are not presented due to its complexity. The range of the fixed transfer payment is $M\in [\frac{\left[\left(E-B+h^{2}\right)\alpha -\left(2h\bar{\theta }-B\right)\right]\alpha AT^{4}-DA}{8\alpha T^{4}\left(A+2c_{f}T^{2}\right)\left(1-\alpha \right)},\frac{\left[\left(E-2B+3h^{2}\right)\alpha -\left(2{\bar{\theta }}^{2}-2B+2h\bar{\theta }-2{c_{f}}^{2}\right)\right]\alpha AT^{4}-3DA}{16\alpha T^{4}\left(A+2c_{f}T^{2}\right)\left(1-\alpha \right)}]$.

**Table 4:** Price, demand, and profit under different modes.

Modes	Pure PYO Mode (PP)	Self-Operated Dual-Channel Mode (SD)	Decentralized Dual-Channel Mode (DD)	Centralized Dual-Channel Mode (CD)	Contractual Cooperation Mode (CC)
Offline price	0.8150000000	0.8150000000	0.8150000000	0.8150000000	0.8150000000
Online price	-	0.6455555555	0.6970833332	0.6455555555	0.6455555555
Wholesale price	-	-	0.6455555555	-	0.0315016900
Revenue sharing ratio	-	-	-	-	0.8957025592
Offline demand	0.7750000000	0.4314814813	0.6032407413	0.4314814813	0.4314814813
Online demand	-	0.4907407410	0.2453703696	0.4907407410	0.4907407410
Total demand	0.7750000000	0.9222222223	0.8486111109	0.9222222223	0.9222222223
Farmer cooperative’s profit	0.6006250000	0.4511985596	0.6259117798	-	[0.6259117796, 0.6385551698]
Online retailer’s profit	-	-	0.0126433899	-	[0.0126433898, 0.0252867801]
Total profit	0.6006250000	0.4511985596	0.6385551697	0.6511985596	0.6511985596

## Appendix A

$A=\left[\left(h+c_{f}-\bar{\theta }\right)\alpha +\bar{\theta }\right]T^{2}-\eta {t_{o}}^{2}$,

$B=4\bar{\theta }+2h^{2}-4h+2$,

$D=2\eta t_{o}{}^{2}T^{2}[(h-\bar{\theta })\alpha +c_{f}+\bar{\theta }]-t_{o}{}^{4}\eta^{2}-2c_{f}\bar{\theta }(1-\alpha )T^{4}-{\bar{\theta }}^{2}T^{4}-c_{f}h\alpha T^{4}$,

$E=2h\bar{\theta }-{\bar{\theta }}^{2}+c_{f}$,

**Proof of Demand Model.** The utility functions of consumers in different shopping modes are

(A1) $$u_{i}=\{\begin{array}{c} \begin{array}{c} \nu \end{array}-p_{f}+\bar{\theta }-h\begin{array}{c} \left(i=f\right) \end{array}\\ \begin{array}{c} \alpha \nu \end{array}-p_{o}+\theta (t_{o})\begin{array}{c} \left(i=o\right) \end{array} \end{array}$$

The corresponding indifference utility is obtained, $\nu_{f}=p_{f}-\bar{\theta }+h$, $\nu_{o}=\frac{p_{o}-\theta (t_{o})}{\alpha }$ and $\nu_{fo}=\frac{p_{f}-p_{o}+\theta (t_{o})-\bar{\theta }+h}{1-\alpha }$. In the pure PYO mode, $Q_{f}=\int \begin{array}{c} 1\\ \nu_{f} \end{array}d\nu =1-p_{f}-\bar{\theta }+h$. In the dual-channel supply chain, when $\nu_{f}\geq \nu_{o}$ and $\nu_{fo}\leq 1$, which can be written as $\bar{\theta }-h+\frac{p_{o}T^{2}-\bar{\theta }T^{2}+\eta t_{o}{}^{2}}{\alpha T^{2}}\leq p_{f}\leq 1-\alpha -h+p_{o}+\frac{\eta t_{o}{}^{2}}{T^{2}}$, the range of ν in the offline channel is $\nu_{fo}\leq \nu \leq 1$ and the corresponding demand function is $Q_{f}=\int \begin{array}{c} 1\\ \nu_{fo} \end{array}d\nu =1-\frac{p_{f}-p_{o}-\frac{\eta t_{o}{}^{2}}{T^{2}}+h}{1-\alpha }$, while the range of ν in online channel is $\nu_{o}<\nu <\nu_{fo}$ and the corresponding demand function is $Q_{fo}=\int \begin{array}{c} \nu_{fo}\\ \nu_{o} \end{array}d\nu =\frac{p_{f}-p_{o}-\frac{\eta t_{o}{}^{2}}{T^{2}}+h}{1-\alpha }-\frac{p_{o}-\bar{\theta }+\frac{\eta t_{o}{}^{2}}{T^{2}}}{\alpha }$. □

**Proof of Propositions 1 and 3.** $\pi_{f}(p_{f},p_{o})=(p_{f}-c_{f})Q_{f}+p_{o}Q_{o}-F$ ($\pi_{f}(p_{f},p_{o})=(p_{f}-c_{f})Q_{f}+p_{o}Q_{o}$) is a strictly differentiable concave function of $\left(p_{f},p_{o}\right)$. The Hessian matrix of $\pi_{f}(p_{f},p_{o})$ about $\left(p_{f},p_{o}\right)$ is $\frac{\partial^{2}\pi_{f}}{\partial^{2}(p_{f},p_{o})}=\left[\begin{array}{cc} -\frac{2}{1-\alpha } & \frac{2}{1-\alpha }\\ \frac{2}{1-\alpha } & -\frac{2}{1-\alpha }-\frac{2}{\alpha } \end{array}\right]$. The principal minors are $-\frac{2}{1-\alpha }<0$ and $\frac{4}{(1-\alpha )\alpha }>0$, and the Hessian matrix is strictly negative. So, π is a strictly concave function. The optimal pricing decision is arrived at after solving the first-order condition $\frac{\partial \pi_{f}}{\partial p_{f}}=\frac{\partial \pi_{f}}{\partial p_{o}}=0$. And we get

(A2) $$\{\begin{array}{c} p_{f}^{s*}=\frac{1}{2}(1+\bar{\theta }+c_{f}-h)\\ p_{o}^{s*}=\frac{1}{2}(\alpha +\bar{\theta }-\frac{\eta t_{o}{}^{2}}{T^{2}}) \end{array}$$

Substitute Equation (A2) into the demand function and profit function, we can get demand and profit of the GASC. According to the constraints of dual-channel in the market of this mode, we know that the condition L1 is $\alpha <1+\bar{\theta }-\theta (t_{o})-h-c_{f}$, and when $\bar{\theta }>h+c_{f}$, $\alpha <\frac{\theta (t_{o})}{\bar{\theta }-h-c_{f}}$. □

**Proof of Proposition 2.** $\frac{\partial^{2}\pi_{o}}{\partial {\left(p_{o}\right)}^{2}}=-\frac{2}{1-\alpha }-\frac{2}{\alpha }<0$, so $\pi_{o}(p_{o})$ is a strictly differentiable concave function. Solving the equation for the first-order condition $\pi_{o}^{\prime }(p_{o})=0$, the optimal decision of pricing is obtained as

(A3) $$p_{o}^{d}(w,p_{f})=\frac{1}{2}\;[\alpha (h+p_{f}-\bar{\theta })+w+\bar{\theta }-\frac{\eta t_{o}^{2}}{T^{2}}]$$

Substituting (A3) in the profit function of the farmer cooperative, $\pi_{f}(p_{f},w,p_{o}^{d}(p_{f},w))$ is obtained. $\pi_{f}(p_{f},w,p_{o}^{d}(p_{f},w))$ is a strictly differentiable concave function of $\left(p_{f},w\right)$. The Hessian matrix of $\pi_{f}(p_{f},w,p_{o}^{d}(p_{f},w))$ with respect to $\left(p_{f},w\right)$ is $\frac{\partial^{2}\pi_{f}}{\partial^{2}(p_{f},w)}=\left[\begin{array}{cc} -\frac{2-\alpha }{1-\alpha } & \frac{2}{(1-\alpha )\alpha }\\ \frac{2}{(1-\alpha )\alpha } & -\frac{1}{1-\alpha }-\frac{1}{\alpha } \end{array}\right]$. The principal minors are $-\frac{2-\alpha }{1-\alpha }<0$ and $\frac{2}{(1-\alpha )\alpha }>0$ with a strictly negative Hessian matrix. So, $\pi_{f}$ is strictly concave. After solving the first-order condition $\frac{\partial \pi }{\partial p_{f}}=\frac{\partial \pi }{\partial w}=0$, the optimal decision $\left(p_{f}^{d},w^{d}\right)$ is obtained. Substituting $\left(p_{f}^{d},w^{d}\right)$ in the equation, and we get the optimal decision of the decentralized dual-channel mode.

(A4) $$\{\begin{array}{c} p_{f}^{d*}=\frac{1}{2}(1+\bar{\theta }+c_{f}-h)\\ p_{o}^{d*}=\frac{1}{4}[\alpha (2+h+c_{f}-\bar{\theta })+3\bar{\theta }-\frac{3\eta t_{o}{}^{2}}{T^{2}}]\\ w^{d*}=\frac{1}{2}(\alpha +\bar{\theta }-\frac{\eta t_{o}{}^{2}}{T^{2}}) \end{array}$$

Substitute Equation (A4) into the demand function and profit function, we can get demand and profit of the GASC. According to the constraints of dual-channel in the market of this mode, we know that the condition L2 is $\alpha <2-\frac{2+\theta (t_{o})}{2+\bar{\theta }-h-c_{f}}$, and when $\bar{\theta }>h+c_{f}$, $\alpha <\frac{\theta (t_{o})}{\bar{\theta }-h-c_{f}}$. □

**Proof of Corollary 1.** From Equations (4), (8) and (14), we know $\pi_{f}^{d*}-\pi_{f}^{b*}=\frac{{\left[\left(h+c_{f}-\bar{\theta }\right)\alpha +\theta \left(t_{o}\right)\right]}^{2}}{8(1-\alpha )\alpha }>0$, $\pi_{f}^{s*}-\pi_{f}^{b*}=\frac{{\left[\left(h+c_{f}-\bar{\theta }\right)\alpha +\theta \left(t_{o}\right)\right]}^{2}}{4(1-\alpha )\alpha }-F$, $\pi_{f}^{s*}-\pi_{f}^{d*}=\frac{{\left[\left(h+c_{f}-\bar{\theta }\right)\alpha +\theta \left(t_{o}\right)\right]}^{2}}{8(1-\alpha )\alpha }-F$. From the design principle of cooperation contract in contractual cooperation mode (Equations (25) and (27)), we can easily know that $Q_{f}^{N*}=Q_{f}^{*}$, $Q_{o}^{N*}=Q_{o}^{*}$, and $\pi^{N*}=\pi^{*}>\pi^{d*}$. And set $G=\frac{{\left[\left(h+c_{f}-\bar{\theta }\right)\alpha +\theta \left(t_{o}\right)\right]}^{2}}{(1-\alpha )\alpha }$. When $0<F<\frac{2}{16}G$ and $\pi_{f}^{N}+M_{\max}<\pi_{f}^{s*}$**,** $\pi_{f}^{s*}>\pi_{f}^{N*}>\pi_{f}^{d*}>\pi_{f}^{b*}$. We can get other conditions likewise. □

**Proof of Corollary 2.** From Equations (4), (8), (14), (17) and (25), we know $\pi_{f}^{s*}-\pi_{f}^{b*}=\frac{{\left[\left(h+c_{f}-\bar{\theta }\right)\alpha +\theta \left(t_{o}\right)\right]}^{2}}{4(1-\alpha )\alpha }-F$, $\pi^{d*}-\pi_{f}^{b*}=\frac{3{\left[\left(h+c_{f}-\bar{\theta }\right)\alpha +\theta \left(t_{o}\right)\right]}^{2}}{16(1-\alpha )\alpha }>0$, $\pi_{f}^{s*}-\pi^{d*}=\frac{{\left[(h+c_{f}-\bar{\theta })\alpha +\theta (t_{o})\right]}^{2}}{16(1-\alpha )\alpha }-F$, $\pi^{*}-\pi_{f}^{s*}=F>0$, $\pi^{*}-\pi^{d*}=\frac{{\left[(h+c_{f}-\bar{\theta })\alpha +\theta (t_{o})\right]}^{2}}{16T^{2}(1-\alpha )\alpha }>0$, $\pi^{*}=\pi^{N*}$. In pure PYO mode and self-operated dual-channel mode, there are only one member that the farmer cooperative. Hence, the profit of GASC in these two modes is equal to the profit of the farmer cooperative, that is $\pi_{f}^{b*}=\pi_{}^{b*}$, $\pi_{f}^{s*}=\pi_{}^{s*}$. When $0<F<\frac{1}{16}G$, $\pi^{*}=\pi^{N*}>\pi_{}^{s*}>\pi^{d*}>\pi_{}^{b*}$. We can get other conditions likewise. □

**Proof of Corollary 3.** From Equations (4), (8), (14), (17) and (25), we know $Q_{o}^{s*}-Q_{o}^{d*}=\frac{\theta \left(t_{o}\right)-\left(\bar{\theta }-h-c_{f}\right)\alpha }{4\alpha (1-\alpha )}$, $Q_{}^{s*}-Q^{b*}=\frac{\left(\bar{\theta }-h-c_{f}\right)\alpha +\theta \left(t_{o}\right)}{2\alpha }$, $Q_{o}^{N*}=Q_{o}^{*}=Q_{o}^{s*}$, $Q^{b*}-Q_{f}^{s*}=\frac{\theta \left(t_{o}\right)-\left(\bar{\theta }-h-c_{f}\right)\alpha }{2(1-\alpha )}$, $Q_{f}^{d*}-Q_{f}^{s*}=\frac{\theta \left(t_{o}\right)-\left(\bar{\theta }-h-c_{f}\right)\alpha }{4(1-\alpha )}$, $Q_{}^{s*}-Q_{}^{d*}=\frac{\left(\bar{\theta }-h-c_{f}\right)\alpha +\theta \left(t_{o}\right)}{4\alpha }$, $Q_{}^{b*}-Q_{f}^{d*}=\frac{\theta \left(t_{o}\right)-\left(\bar{\theta }-h-c_{f}\right)\alpha }{4(1-\alpha )}$, $Q_{}^{d*}-Q_{}^{b*}=\frac{\theta \left(t_{o}\right)-\left(\bar{\theta }-h-c_{f}\right)\alpha }{4\alpha }$, $Q_{f}^{N*}=Q_{f}^{*}=Q_{f}^{s*}$, $Q_{}^{d*}-Q_{f}^{s*}=\frac{\left[\theta \left(t_{o}\right)-\left(\bar{\theta }-h-c_{f}\right)\alpha \right]\left(1+\alpha \right)}{4\alpha (1-\alpha )}$, $Q_{}^{s*}-Q_{f}^{d*}=\frac{\left[\theta \left(t_{o}\right)-\left(\bar{\theta }-h-c_{f}\right)\alpha \right]\left(2-\alpha \right)}{4\alpha (1-\alpha )}$. $Q_{}^{N*}=Q_{}^{*}=Q_{}^{s*}$. When $\bar{\theta }>h+c_{f}$, $Q^{s*}=Q^{*}=Q^{N*}>Q_{}^{d*}>Q^{b*}>Q_{f}^{d*}>Q_{f}^{s*}=Q_{f}^{*}=Q_{f}^{N*}$. We can get other conditions likewise. □

**Proof of Corollary 4.** From Equations (4), (8), (14), (17) and (25), we know $p_{f}^{b*}=p_{f}^{s*}=p_{f}^{d*}=p_{f}^{*}=p_{f}^{N*}$, $p_{o}^{s*}=p_{o}^{*}=p_{o}^{N*}=w_{}^{d*}$, $p_{o}^{s*}-p_{o}^{d*}=\frac{\theta (t_{o})-(\bar{\theta }-h-c_{f})\alpha }{4}>0$. □
